# Reassessing Design and Analysis of two-Colour Microarray Experiments Using Mixed Effects Models

**DOI:** 10.1002/cfg.464

**Published:** 2005-04

**Authors:** Guilherme J. M. Rosa, Juan P. Steibel, Robert J. Tempelman

**Affiliations:** 1 Department of Animal Science Michigan State University East Lansing MI USA; 2 Department of Fisheries and Wildlife Michigan State University East Lansing MI USA; 3 Department of Animal Science 1205-I Anthony Hall Michigan State University East Lansing MI 48824-1225 USA

## Abstract

Gene expression microarray studies have led to interesting experimental design and statistical analysis challenges. The comparison of expression profiles across populations is one of the most common objectives of microarray experiments. In this manuscript we review some issues regarding design and statistical analysis for two-colour microarray platforms using mixed linear models, with special attention directed towards the different hierarchical levels of replication and the consequent effect on the use of appropriate error terms for comparing experimental groups. We examine the traditional analysis of variance (ANOVA) models proposed for microarray data and their extensions to hierarchically replicated experiments. In addition, we discuss a mixed model methodology for power and efficiency calculations of different microarray experimental designs.


**Comparative and Functional Genomics**
*Comp Funct Genom* 2005; **6**: 123–131.
Published online in Wiley InterScience (www.interscience.wiley.com). **DOI:** 10.1002/cfg.464

**Conference Paper**

### Reassessing design and analysis of two-colour microarray experiments using mixed effects models

Guilherme J. M. Rosa[1,2*], Juan P. Steibel[1] and Robert J. Tempelman[1]
[1]*Department of Animal Science, Michigan State University, East Lansing, MI, USA*
[2]*Department of Fisheries and Wildlife, Michigan State University, East Lansing, MI, USA*

**Correspondence to:
Guilherme J. M. Rosa,
Department of Animal Science,
1205-I Anthony Hall, Michigan
State University, East Lansing, MI
48824-1225, USA.
E-mail: rosag@msu.edu*

Received: 19 January 2005
Accepted: 3 February 2005

### Abstract

**Gene expression microarray studies have led to interesting experimental design and statistical analysis challenges. The comparison of expression profiles across populations is one of the most common objectives of microarray experiments. In this manuscript we review some issues regarding design and statistical analysis for two-colour microarray platforms using mixed linear models, with special attention directed towards the different hierarchical levels of replication and the consequent effect on the use of appropriate error terms for comparing experimental groups. We examine the traditional analysis of variance (ANOVA) models proposed for microarray data and their extensions to hierarchically replicated experiments. In addition, we discuss a mixed model methodology for power and efficiency calculations of different microarray experimental designs.** **Copyright © 2005 John Wiley & Sons, Ltd.**

**Keywords: microarray; hierarchical replication; mixed models; experimental design; group comparison**

### Introduction

Early applications of the two-colour microarray technology were generally limited to experiments with a single slide comparing two mRNA samples, e.g. treated and control samples. Numerous statistical approaches were suggested for selecting differentially expressed genes between the two target samples. Some methods considered spots as the experimental units, e.g. when genes are spotted multiple times on each array; others employed different sorts of shrinkage estimation to come up with estimates of variances and test statistics. Biological variability, however, was not yet accounted for in those experiments and statistical procedures. As a result, inferences obtained from those early experiments, such as differential expression significance, as well as gene expression fold change estimates, were restricted to the two specific samples used in the competitive hybridization. Broader inferences, relative to the two populations of interest, were not possible (albeit researchers have frequently ignored this fact), simply because of the lack of replication.

The importance of replication in gene expression experiments is nowadays unambiguous. With two-colour platforms (cDNA or long oligonucleotide arrays), replication is considered at various hierarchical levels, including multiple subjects per experimental group (biological replication), as well as multiple slides per subject or multiple spots per gene (technical replication). Multiple spots per gene are intended to attenuate spatial effects on each slide. Multiple slides per mRNA sample are generally suggested to attenuate technical noise. Only biological replication, however, can provide information on subject-to-subject variability, essential for inferring differences on the populations represented in the experiment.

This new perception regarding microarray experiments has given rise to further experimental

Copyright © 2005 John Wiley & Sons, Ltd.

design and statistical analysis challenges. From an experimental design perspective, planning a microarray trial now involves deciding not only the number of replications (at both technical and biological levels), but also the distribution of the mRNA samples across slides and the labelling assignments of mRNA samples in each competitive hybridization.

From a data analysis standpoint, after a microarray experiment is conducted and the image analysis and data normalization are performed (which are *per se* very interesting and important steps of any microarray study), statistical tools are needed to deal with data sets of unprecedented complexity and dimensionality. Various statistical procedures have been proposed or adapted from traditional methods for the analysis of replicated microarray experiments. Available procedures span a broad range of statistical tools, such as linear and nonlinear models, alternative distributional assumptions, multivariate methods, shrinkage estimators, multiple testing significance level adjustments and so on.

Specifically for the comparison of expression profiles across groups or populations (within either experimental or observational settings), the ANOVA models (including those with random effects) are the most popular, due to their flexibility and ease of use, as well as the availability of software for their implementation. A careful look at recent publications making use of such models for microarray data analysis, however, reveals that the distinction between the different levels of replications is not fully appreciated by a number of researchers, either when performing $F$ tests for the selection of differentially expressed genes, or for precision and power analysis before the experiments are conducted. As a result, sample size calculations and test statistics $p$-values are unavoidably incorrect, and so is any *a posteriori* multiple testing approach for significance adjustment based on these misleading $p$-values. Therefore, the accurate definition of the experimental unit, with the appropriate distinction between technical and biological replication, is crucial for the validity of inferences from microarray gene expression studies. In this context, the linear mixed effects models play a central role. These models are extremely useful for the analysis of data from a wide range of experimental design settings, including incomplete block structures, missing data and different hierarchical levels of replication and co-variance structures, such as those encountered in microarray experiments.

In this manuscript, a brief overview of incomplete block designs and their application to two-colour microarray experiments is presented. The traditional analysis of variance (ANOVA) models proposed for microarray data are reviewed, as well as their extensions to mixed effects models to deal with hierarchically replicated experiments. In addition, we discuss how these models can be used to compare different experimental designs, as well as to assess precision and power. A final section provides some concluding remarks and directions for future research.

### Alternative design layouts for microarray experiments

### Incomplete block structures

A first step of planning a microarray experiment refers to the design of the array, including the choice of the clones to be represented, the number of spots and their spatial distribution on the slide. A second step relates to the allocation of mRNA samples to the slides, as well as the assignment of labelling tags. Whenever an experiment involves only two experimental groups, a natural alternative for the allocation of the samples to the arrays is to have one sample from each group represented in each slide. Some additional variations may be considered as well, including dye-swap and pooling of samples. However, if more than two groups are to be compared, an incomplete block structure is inevitable and a number of different experimental layouts may be adopted.

To review the concept of completely randomized and block designs, consider the four fictitious agronomical experiments depicted in Figure 1, involving three crop varieties (A, B and C) and six plots. Squares with different border shadings represent heterogeneous plots, due for example to differences in soil fertility, water and light abundance, and so on. In the case where plots are homogeneous (Figure 1a), each variety can be assigned to two of the plots with no restriction on the randomization, within a completely randomized design. On the other hand, if there are any inherent factors splitting the plots into blocks, such that plots are homogeneous within blocks but may be heterogeneous across blocks, the randomization should

Copyright © 2005 John Wiley & Sons, Ltd. 

be performed within blocks, such that varieties are spread out across blocks. This restriction in the randomization generates the so-called block design. If the number of plots within each block is equal to the number of varieties, each variety will be represented in each block, within a complete block design (Figure 1b). However, if it is not possible to allocate all varieties in each block, an incomplete block structure is required. An alternative to overcome this situation is to introduce an additional variety in the experiment, which is assigned to every block of the experiment as a mean to estimate and correct for differences among blocks. This is the so-called reference design (Figure 1c1). Another possibility to deal with this situation is to assign different sets of varieties to each plot having, for example, any pair of varieties appearing together equally often within some block (Figure 1c2).

An important concept regarding incomplete block designs refers to direct and indirect comparisons. A direct comparison between two varieties is possible only if the varieties are represented within the same block. An example in Figure 1 would be the comparison of the varieties A and B within the first block (white border plots) of Figure 1c2. An indirect comparison refers to a contrast between varieties represented in different blocks, but having a third variety that links the two blocks, making it possible to correct for differences due to plots heterogeneity. Examples of indirect comparisons would be any contrast between the varieties A, B or C on the reference design of Figure 1c1. Another example would be the comparison of varieties A and C on the two first blocks (white and hatched border plots) of Figure 1c2, in which case the variety B would work as the linking variety.

Microarray experiments resemble the situations illustrated in Figures 1c1 and 1c2, where varieties represent the mRNA samples, and the blocks represent the slides, which can accommodate two samples, one with each dye labelling. Different incomplete block designs have been discussed in the literature for microarray experiments, including layouts such as those of Figure 1c1, in which a reference mRNA sample is obtained for example by pooling target samples or by using genomic DNA, and of Figure 1c2, with the so-called loop (or circular) designs (Kerr and Churchill, 2001a). Some advantages and disadvantages of each alternative design have been discussed, e.g. by Kerr and Churchill (2001a) and Yang and Speed (2002). Direct comparisons provide more information regarding a specific contrast between two samples, as compared to indirect comparisons. The estimated variance for a specific contrast results from the combination of all direct and indirect comparisons linking the two varieties in the experiment.

### Graphical representation of microarray experiments

Microarray experiments are generally illustrated by a set of arrows (Yang and Speed, 2002), in which each arrow connects the two samples that are hybridized together in a single slide. The arrow's tail and head denote the Cy3 (green) and Cy5 (red) labelling assignments, respectively. A number on the top of an arrow denotes the number of replications for that specific hybridization. For example, Figure 2a depicts three replicated hybridizations of samples A and B, which are labelled with Cy3 and Cy5, respectively. Figure 2b and 2c represent the reference and loop designs discussed previously, respectively. In the reference experiment portrayed, two target samples (A and B) are hybridized twice with a third common sample (R). In the loop experiment, the three target samples are arranged on

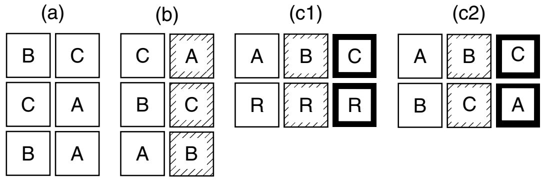

**Figure 1.** Four possible design layouts for an agronomical experiment involving three crop varieties (A, B and C) and six plots. Completely randomized (a) and complete block (b) designs, and incomplete block structures: reference (c1) and circular (c2) designs

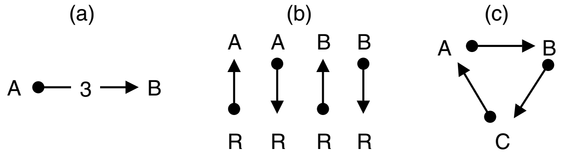

**Figure 2.** Graphical representation of two-colour microarray experiments. (a) Three replicated hybridizations of samples A and B, labelled with Cy3 and Cy5, respectively. (b) Reference design with two target samples (A and B). (c) Loop experiment with three target samples and three slides

Copyright © 2005 John Wiley & Sons, Ltd.

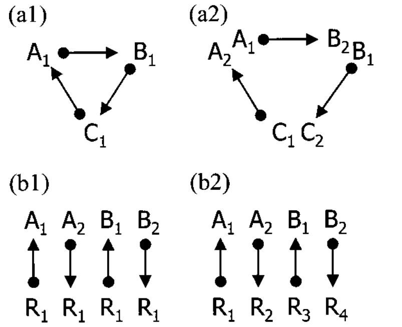

**Figure 3.** A more appropriate graphical representation of two-colour microarray experiments, with indexes representing biological replications. (a1, a2) Connected and classical loop designs, respectively. (b1, b2) Common and classical reference designs, respectively three slides, so that each sample is represented on two slides, with both labelling dyes.

In the context of different levels of replication, however, this simplified graphical representation is not totally satisfactory, e.g. Figure 2c does not make clear whether the two samples of group A refer to a single mRNA sample that was split and labelled with different dyes, or to mRNA samples from two different subjects. These two completely different scenarios are much better illustrated by Figure 3a1 and 3a2, respectively. The scenario in Figure 3a1 is referred to as connected loop design, whereas the scenario in Figure 3a2 consists of a traditional incomplete block design.

Likewise, the reference design depicted in Figure 2b can represent either the so-called common reference design (Figure 3b1) or the classical reference design (Figure 3b2) generally found in the agronomical literature. These seemingly small differences can make a huge difference to experimental design and statistical modelling of microarray data, as discussed in the sections below.

### Linear models for microarray data

### ANOVA models

The first use of an ANOVA approach for analysing microarray fluorescence intensities was presented by Kerr *et al.* (2000). Their model may be described as:

$$y_{adgvr} = \mu + A_a + D_d + (AD)_{ad} + G_g + (AG)_{ag} + (DG)_{dg} + (VG)_{vg} + \varepsilon_{adgvr} \quad (1)$$

where $y_{adgvr}$ represents the expression intensities on the log scale; $\mu$ is an overall constant; $A_a$, $D_d$ and $(AD)_{ad}$ are 'global factors' that account for variation between arrays and dyes; $G_g$ are the gene effects; $(AG)_{ag}$ represents array by gene interactions; $(DG)_{dg}$ are gene specific dye effects; $(VG)_{vg}$ are the quantities of interest, related to differential expression of gene $g$ specifically attributable to variety (treatment) $v$; and $\varepsilon_{adgvr}$ are random residual terms with variance $\sigma^2_\varepsilon$. This paper was an important contribution to the microarray data analysis literature, as it proposed that multiple factors (such as dye, slides, patches within slides and so on) could be accounted for simultaneously in the analysis. Nowadays, however, some important drawbacks of this modelling approach are recognized. First, a common residual variance is considered for all genes; it is now well known that this is a very strong and unrealistic assumption. Second and even more important, a fixed-effects model is adopted, ignoring the multiple sources of random variation, and considering the spot as the 'fundamental experimental unit' (Kerr and Churchill, 2001b).

### Biological and technical replication

There are multiple sources of variability in microarray experiments, and an important distinction between two specific components. One is related to biological or between-subjects variation (i.e. individual-to-individual variability) and the other refers to within-subject variability, related to multiple measurements on the same subject. This latter source represents measurement error or subsampling variability, e.g. due to differential efficiency of array hybridizations and image analysis. Because of biological variability in gene expression due to genetic and environmental differences among subjects, it is fundamentally important to compare treatments based on different individuals per treatment. It is only the number of subjects per treatment that determines true replication, as opposed to pseudoreplication based on multiple measurements or subsamples per subject (Gill, 1978).

To attenuate measurement error, researchers are often interested in multiple measures for each individual. Different levels of technical replication are possible, including multiple tissue samples from each subject, multiple RNA extractions and cDNA syntheses from each tissue sample, repeated hybridizations of each cDNA sample on different arrays, multiple spots of the same gene spread across each array, and several image analyses of each array. Whenever multiple levels of replication are considered, a hierarchical modelling approach is required for sound statistical inference on treatment differences.

### Mixed effects models

Wolfinger *et al.* (2001) extended the ANOVA approach of Kerr *et al.* (2000), including random effects to model the dependence among observations relative to the same spots or arrays, and allowing for gene-specific variance components. For computational convenience, a two-step approach was proposed. The first stage, referred to as 'global normalization', fits all the data using a model expressed as:

$$y_{adgvr} = \mu + A_a + D_d + (AD)_{ad} + e_{adgvr}$$

where the terms are defined as for model (1). From the global normalization, the estimated residuals $\hat{e}_{adgvr}$ are saved. The second stage comprises a series of gene specific models, which can be written as:

$$\hat{e}_{adgvr} = \mu_g + A_{ag} + D_{dg} + V_{vg} + \varepsilon_{adgvr} \quad (2)$$

where $\mu_g$, $A_{ag}$, $D_{dg}$, and $V_{vg}$ are gene-specific overall constant, array effects, dye effects and treatment effects, respectively. Gene-specific variance components are fitted for the random effects of arrays and the residual terms. Additional factors may be included into the model, depending on the treatment structure (e.g. factorial experiments) and design settings (e.g. patch effects, spot effects and interactions involving random factors). For a discussion on the choice of fixed and random effects in microarray experiments, see e.g. Steibel *et al.* (2005).

It is important to note, however, that it does not suffice to include biological replication in the experiment and to include random effects into the model; a correct definition of the experimental unit is also crucial, with an appropriate characterization of the model as well as the denominator of the ANOVA $F$-statistic for comparing treatments (Churchill, 2002; Wernisch *et al.*, 2003).

### Connection between model choice and design

The statistical modelling of experimental data should be intimately connected with the design layout of the experiment. To illustrate this point, consider a simple experiment where two treatments (varieties) are compared, and $n$ subjects within each treatment are measured $m$ times for a certain trait. Assume that the data generation process can be described as:

$$y_{vsr} = \mu + V_v + S(V)_{vs} + \varepsilon_{vsr} \quad (3)$$

where $y_{vsr}$ is the observed trait; $\mu$ is a general constant; $V_v$ represents the treatment effects; $S(V)_{vs}$ refers to the random effect of subjects within treatments, having variance $\sigma_S^2$; and $\varepsilon_{vsr}$ is a residual term with variance $\sigma_\varepsilon^2$.

Suppose, however, that the following model is used for analysing the data:

$$y_{vsr} = \mu + V_v + e_{vsr} \quad (4)$$

where $e_{vsr}$ is a random term with variance $\sigma_e^2$, assumed to be common to all observations, regardless of whether or not they refer to the same subjects (repeated measurements). Under these circumstances, as the hierarchical replication structure is ignored in the analysis and the ANOVA shown in Table 1 is obtained.

Another incorrect specification for the analysis would be using the model (3), but considering all the components as fixed. In this case, the ANOVA shown in Table 2 would be obtained.

It is clear that the treatment comparisons obtained from such analyses are incorrect, as in both cases the residual mean square would be used as the denominator of the $F$ test. Under the null hypothesis, the $F$ test statistic would tend to be greater than 1, increasing the type I error rate beyond the nominal significance level set by the researcher.

**Table 1.** ANOVA table associated with model (4), when model (3) represents the true data generation process

| SV | DF | E[MS]\|model | E[MS]\|data generation |
|---|---|---|---|
| V | 1 | $\sigma_e^2 + \phi_V$ | $\sigma_\varepsilon^2 + m\sigma_S^2 + \phi_V$ |
| Residual | $2(nm - 1)$ | $\sigma_e^2$ | $\sigma_\varepsilon^2 + \left[\frac{m(n-1)}{nm-1}\right]\sigma_S^2$ |

SV, source of variation; DF, degrees of freedom; E[MS]|model, expected mean squares under model (4); E[MS]|data generation, expected mean squares under model (3).

Copyright © 2005 John Wiley & Sons, Ltd. 

**Table 2.** ANOVA table associated with a fixed effects version of model (3)

| SV | DF | E[MS]\|model | E[MS]\|data generation |
|---|---|---|---|
| V | 1 | $\sigma_\varepsilon^2 + \phi_V$ | $\sigma_\varepsilon^2 + m\sigma_S^2 + nm\phi_V$ |
| S(V) | $2(n-1)$ | $\sigma_\varepsilon^2 + \phi_{S(V)}$ | $\sigma_\varepsilon^2 + m\sigma_S^2$ |
| Residual | $2n(m-1)$ | $\sigma_\varepsilon^2$ | $\sigma_\varepsilon^2$ |

SV, source of variation; DF, degrees of freedom; E[MS]|model, expected mean squares under a fixed effects model; E[MS]|data generation, expected mean squares under model (3).

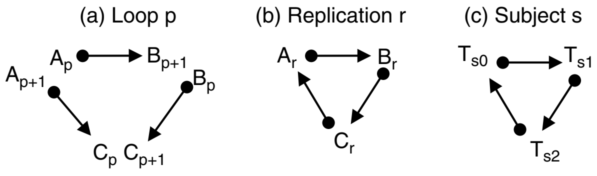

**Figure 4.** The graphics represent the smallest part of alternative circular design layouts. In (a), independent samples are used on each array (classical incomplete block); in (b), each sample is represented on two arrays (connected loop); and in (c), the same subject is assayed on all arrays, such as in a longitudinal study

Similarly to the example discussed above, the statistical modelling of a microarray data should be intimately connected with its experimental design. If a fixed effects model is adopted, the number of differentially expressed genes may be overestimated. This is because when a fixed effects model is used, the error term refers only to the lowest level of replication (e.g. spots). This apparent increase in power, however, translates into an inflation of the type I error and false discovery rates.

To illustrate some possible mixed effects models and their connection with the design layout of microarray studies, consider the three experiments depicted in Figure 4. The model (2) described above would be a reasonable choice for analysing data on each gene from the experiment shown in Figure 4a, in which different subjects are represented in each array. In this case, observations from different slides may be regarded as independent, and the random effect of arrays models the co-variance among observations within each slide. Moreover, if each gene is represented on multiple spots per slide, an additional term (the random effect of spots within arrays) is needed to model this extra level of technical replication.

Model (2), however, is not unique and may be not appropriate to model microarray data from other design layouts; e.g. if multiple connected loop structures, such as the one represented in Figure 4b, are considered, the dependency between observations relative to the same subject on different arrays should be included in the model. This may be accomplished by including a term for the random effect of subjects within treatments, as follows:

$$\hat{e}_{adgvsr} = \mu_g + A_{ag} + D_{dg} + V_{vg} + S(V)_{svg} + \varepsilon_{adgvsr}$$

It is important to note that in this case the term $S(V)_{svg}$ is the appropriate error term (denominator of the F test) to compare treatments.

As another example, consider an experiment in which each subject is evaluated under different conditions. Suppose, for example, that a blood sample from each subject is split and submitted to three different treatments ($T_0$, $T_1$ and $T_2$), before the evaluation of gene expression. The three samples from each subject are then assayed using a loop structure (Figure 4c). Under these circumstances, all observations within a specific loop may present some level of dependence. A possible model for the analysis of such data may be described as:

$$\hat{e}_{adgvsr} = \mu_g + D_{dg} + V_{vg} + S_{sg} + VS_{vsg} + A(S)_{asg} + \varepsilon_{adgvsr}$$

where $S_{sg}$ represents the random effect of subjects, $VS_{vsg}$ is the interaction between treatments and subjects, and $A(S)_{asg}$ refers to the random effect of arrays within loops (subjects). In this case, the error term for the comparison of treatments is the interaction $VS_{vsg}$.

### Relative efficiency and robustness of alternative experimental designs

A number of studies addressed specific aspects of microarray experimental design, e.g. Lee and Whitmore (2002) studied the sample size and power calculation for replicated arrays; Black and Doerge (2003) determined the optimal number of replicated spots; and Pavlidis *et al.* (2003) evaluated the effect of biological replication. Kerr and Churchill (2001b), Dobbin and Simon (2002) and Yang and Speed (2002) compared several designs in terms

Copyright © 2005 John Wiley & Sons, Ltd. 

of the relative efficiency to estimate a treatment effect. More recently, Churchill (2002) and Cui and Churchill (2003) addressed the problem of hierarchical replication and presented general expressions for the variances of treatment contrasts, and Kerr (2003) compared three common designs using a mixed model ANOVA. A more explicit use of mixed models methodology for comparing microarray experimental designs was discussed by Tempelman (2005) and Steibel *et al.* (2005). They considered the method proposed by Stroup (2002) to determine power, standard errors and sample size calculations. This methodology is simple, as it does not require stochastic simulations and can be implemented using standard mixed models analysis software. A brief overview of this methodology is provided below.

A linear mixed effects model can be expressed in a matrix notation as $\mathbf{y} = \mathbf{X}\boldsymbol{\beta} + \mathbf{Zu} + \mathbf{e}$, where $\mathbf{y}$ is a vector of observations, representing in our case the expression intensities in the log scale. The vectors $\boldsymbol{\beta}$ and $\mathbf{u}$ represent fixed and random effects in the model, such as the treatment and slides effects, respectively; and $\mathbf{X}$ and $\mathbf{Z}$ are matrices of constants associate with $\boldsymbol{\beta}$ and $\mathbf{u}$, which characterize the experimental settings, such as the treatment structure and the experimental design, respectively. It is generally assumed that $\mathbf{u}$ and $\mathbf{e}$ have independent multivariate normal distributions with mean vectors $\mathbf{0}$ and co-variance matrices $\mathbf{G}$ and $\mathbf{R}$.

The comparison of treatment groups involves hypothesis testing of estimable functions of the form $\mathbf{K}'\boldsymbol{\beta}$, where $\mathbf{K}$ is a matrix of constants. An approximated $F$ statistic for testing the hypothesis $H_0 : \mathbf{K}'\boldsymbol{\beta} = \mathbf{0}$ (e.g. no differential expression among treatments) is given by:

$$F(\mathbf{K}'\boldsymbol{\beta} = \mathbf{0}) = \frac{\hat{\boldsymbol{\beta}}'\mathbf{K}(\mathbf{K}'\hat{\mathbf{C}}\mathbf{K})^{-1}\mathbf{K}'\hat{\boldsymbol{\beta}}}{Rank(\mathbf{K})}$$

where $\hat{\boldsymbol{\beta}} = (\mathbf{X}'\mathbf{V}^{-1}\mathbf{X})^{-}\mathbf{X}'\mathbf{V}^{-1}\mathbf{y}$, $\mathbf{C} = (\mathbf{X}'\mathbf{V}^{-1}\mathbf{X})^{-}$ and $\mathbf{V} = \mathbf{ZGZ}' + \mathbf{R}$. This statistic has an approximate $F_{[Rank(\mathbf{K}),\nu,\phi]}$ distribution with $Rank(\mathbf{K})$ numerator degrees of freedom; $\nu$ denominator degrees of freedom, e.g. approximated by:

$$\nu = 2E[(\mathbf{K}'\mathbf{CK})]^2/V[(\mathbf{K}'\mathbf{CK})];$$

and non-centrality parameter

$$\phi = \boldsymbol{\beta}'\mathbf{K}(\mathbf{K}'\mathbf{CK})^{-1}\mathbf{K}'\boldsymbol{\beta}$$

The procedure to evaluate power for a given design (which determines $\mathbf{X}$ and $\mathbf{Z}$), co-variance structure (given by $\mathbf{G}$ and $\mathbf{R}$) and treatment differences (encapsulated within $\boldsymbol{\beta}$), is as follows (Stroup, 2002):

1. Determine the critical value ($F_{crit}$) of $F$ needed to reject $H_0 : \mathbf{K}'\boldsymbol{\beta} = \mathbf{0}$, such that $\Pr[\mathrm{F}_{[\mathrm{Rank}(\mathbf{K}),\nu,\phi=0]} > \mathrm{F}_{\mathrm{crit}}] = \alpha$.
2. Determine $\phi$ from $\mathbf{X}$ and $\mathbf{Z}$ that follow from the design, and the assumed $\mathbf{V}$ and $\boldsymbol{\beta}$.
3. Determine the power, that is, $\Pr[\mathrm{F}_{[\mathrm{Rank}(\mathbf{K}),\nu,\phi]} > \mathrm{F}_{\mathrm{crit}}]$.

This procedure was used by Tempelman (2005) to assess power and relative efficiency, as well as robustness to missing arrays or spots, of reference and non-reference layouts, such as those illustrated on Figure 3. The results showed that, for a fixed number of slides, the relative performance of non-reference designs generally exceeds that of reference designs. In addition, the classical loop structure is generally better than the connected loop alternative, whereas the common reference design outperformed the classical reference alternative. Nevertheless, the magnitude of the differences depends on the ratio of biological to technical variability. A similar approach was used by Steibel *et al.* (2005), who compared the loop, dye-swap and reference designs for experiments with two treatments and three levels of replication (subjects, arrays and spots). The reference design was again outperformed by the non-reference alternatives.

It is important to mention, however, that the choice of experimental design and the determination of the sample size in microarray experiments are not exclusively a statistical issue, but they also depend on logistic limitations of each laboratory and field of research. For example, if limited quantities of RNA are available from the test samples, the reference design may be preferred, as the dye swap and the loop designs would require twice the quantity from each subject.

### Discussion and concluding remarks

The mixed effects model approach discussed above performs independent analyses for each gene, one at a time. The drawback of this approach is that, because sample sizes for each gene are usually

Copyright © 2005 John Wiley & Sons, Ltd. 

relatively small, the power to detect differentially expressed genes may be low. Various alternatives have been proposed to overcome this situation and to improve power. Shrinkage estimators aiming to borrow information across genes are a promising alternative in this context. An especially suitable approach for mixed model analysis of microarray data was presented by Feng *et al.* (2005). Their procedure starts with the gene-specific analyses, as discussed above, from which the estimated variance components are transformed to ANOVA components. The distribution of each ANOVA component is then used as a prior distribution in a second analysis, within an empirical Bayes procedure.

Another interesting issue related to microarray data analysis refers to the multiple testing problem. The effect of treatments or experimental conditions is tested for a series of genes, and a specific gene is declared differentially expressed if its $p$-value is less than $\alpha$. The problem with this approach is that, in a situation under the null hypothesis (i.e. no differential expression at all, such as in self-self hybridizations), $100\alpha\%$ of the genes are expected to have significant $p$-values. So, for example, if 100 genes are tested considering $\alpha = 0.05$, five false positives (genes with $p$-values less than 0.05) are expected. In addition, the probability of at least one false positive is $1-0.95^{100} = 0.9941$. The situation evidently gets worse as the number of genes increases. Various approaches have been suggested to adjust the $p$-values and to control family-wise error rates or false discovery rates. For a review on this topic, see e.g. Dudoit *et al.* (2003).

This paper reviews power analysis and sample size calculations for microarray experiments with different sources of variation and hierarchical replication. The calculations require the input of variance components values, as well as of expression fold changes. Plausible values for these quantities can be obtained, for example, from the empirical distribution of parameter estimates obtained in previous experiments. Nonetheless, genes present different values of fold change and variance components, such that any specific value used for sample size calculations will overestimate the power for some genes and underestimate the power for others. Moreover, in a multiple testing scenario, power may be defined in different ways, such as the probability of detecting at least one (or a pre-specified proportion) of the differentially expressed genes. Alternatively, power may be considered in the context of expected discovery rate (EDR), as discussed by Gadbury *et al.* (2004).

### References

Black MA, Doerge RW. 2002. Calculation of the minimum number of replicate spots required for detection of significant expression fold change in microarray experiments. *Bioinformatics* **18**: 1609–1616.

Churchill GA. 2002. Fundamentals of experimental design for cDNA microarrays. *Nature Genet* **32**: 490–495.

Cui X, Churchill GA. 2003. Methods in microarray data analysis III. In *Methods of Microarray Data Analysis III: Papers from Camda '02*, Johnson KF, Lin SM (eds). Kluwer Academic: Dordrecht.

Dudoit S, Shaffer JP, Boldrick JC. 2003. Multiple hypothesis testing in microarray experiments. *Statist Sci* **18**: 71–103.

Dobbin K, Simon R. 2002. Comparison of microarray designs for class comparison and class discovery. *Bioinformatics* **18**: 1438–1445.

Dobbin K, Shih JH, Simon R. 2003. Statistical design of reverse dye microarrays. *Bioinformatics* **19**: 803–810.

Feng S, Wolfinger RD, Chu TM, Gibson GC, McGraw LA. 2005. Empirical Bayesian analysis of variance component models for microarray data. *J Agric Biol Environ Stat* (in press).

Gadbury GL, Page GP, Edwards J, *et al.* 2004. Power and sample size estimation in high dimensional biology. *Statist Methods Med Res* **13**: 325–338.

Gill JL. 1978. *Design and Analysis of Experiments in the Animal and Medical Sciences*, vol I. Iowa State University Press: Ames, IA.

Kerr MK. 2003. Design considerations for efficient and effective microarray studies. *Biometrics* **59**: 822–828.

Kerr MK, Churchill GA. 2001a. Experimental design for gene expression microarrays. *Biostatistics* **2**: 183–201.

Kerr MK, Churchill GA. 2001b. Statistical design and the analysis of gene expression microarray data. *Genet Res* **77**: 123–128.

Kerr MK, Martin M, Churchill GA. 2000. Analysis of variance for gene expression microarray data. *J Comp Biol* **7**: 819–837.

Lee M-LT, Whitmore GA. 2002. Power and sample size for DNA microarray studies. *Statist Med* **21**: 3543–3570.

Leil TA, Ossaditchi A, Nichols TE, Leahey RM, Smith D. 2003. Genes regulated by learning in the hippocampus. *J Neuroci Res* **71**: 763–768.

Pavlidis P, Li QH, Noble WS. 2003. The effect of replication on gene expression microarray experiments. *Bioinformatics* **19**: 1620–1627.

Steibel JP, Tempelman RJ, Rosa GJM. 2005. Power and sample size determinations for two colour microarray experiments based on different levels of replication (submitted for publication).

Stroup WW. 2002. Power analysis based on spatial effects mixed models: a tool for comparing design and analysis strategies in the presence of spatial variability. *J Agric Biol Environ Statist* **7**: 491–511.

Tempelman RJ. 2005. Assessing statistical precision, power, and robustness of alternative experimental designs for two colour

Copyright © 2005 John Wiley & Sons, Ltd. 

microarray platforms based on mixed effects models. *Vet Immunol Immunopathol* (in press).
Wernisch L, Kendall SL, Soneji S, *et al*. 2003. Analysis of whole-genome microarray replicates using mixed models. *Bioinformatics* **19**: 53–61.
Wolfinger RD, Gibson G, Wolfinger ED, *et al*. 2001. Assessing gene significance from cDNA microarray expression data via mixed models. *J Comp Biol* **8**: 625–637.
Yang YH, Speed T. 2002. Design issues for cDNA microarray experiments. *Nature Rev Genet* **3**: 579–588.

Copyright © 2005 John Wiley & Sons, Ltd. 
